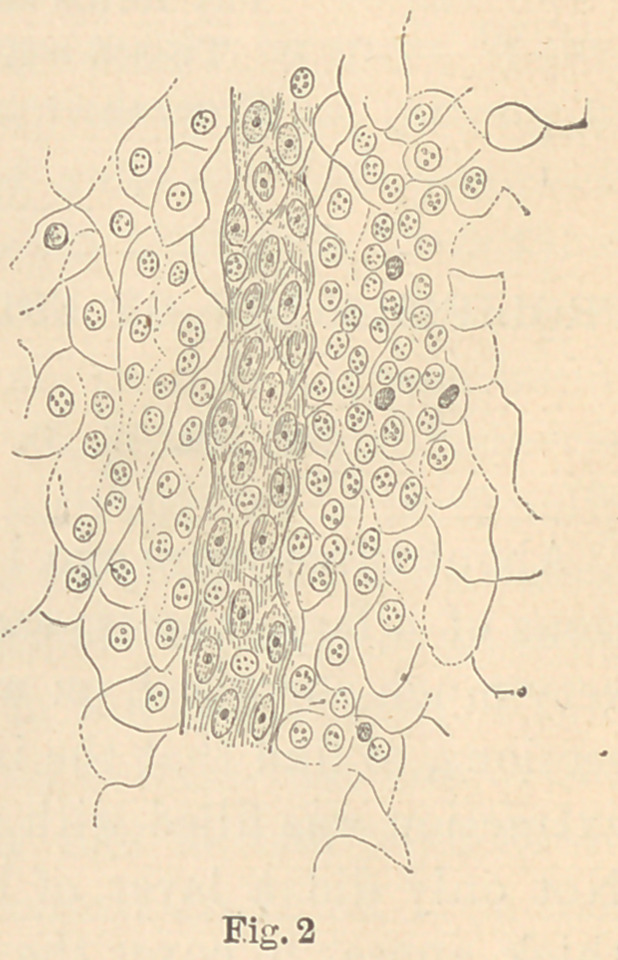# Proliferation of Epithelium in an Alveolar Abscess

**Published:** 1885-01

**Authors:** W. D. Miller

**Affiliations:** Berlin, Germany


### PROLIFERATION OF EPITHELIUM IN AN ALVEOLAR ABSCESS.

BY DR. W. D. MILLER, BERLIN, GERMANY.

About three years ago I had occasion to prepare microscopic sections of a large abscess-sac from an upper bicuspid tooth, and was very much surprised, on making a microscopic examination of the sections, to find that the interior of the sac, which at the time of extraction was filled with pus, had a complete lining of epithelium. Not only did a layer of epithelial tissue, from two to eight cells thick, appear to cover the entire inner surface of the sac, but it sent sprouts into the tissue of the sac, thus forming a network of epithelial tissue, the meshes of which were filled with a reticular connective tissue, infiltrated with round-cells (pus corpuscles).

Fig. 1

In figure one is given a, in part schematic, representation of the appearance of a section under low power; *a*, the inner surface of the sac, and *b*, the lining of epithelium, which at *c* has already formed a network in the substance of the sac, and at *d* is sending a new sprout into the same.

In figure two is seen a small portion of the same under higher power.

Two interesting questions arise in connection with this case; first, how was the epithelium produced within the abscess? second, what effect could it have had upon the course of the disease?

In respect to the first of these questions, the same principle holds true for epithelial cells as for cells in general; that is, epithelial tissue can be produced only by proliferation of previously existing epithelium; it cannot, therefore, have arisen by a transformation of any of the elements ordinarily found in an alveolar abscess, but must in all probability have entered the abscess from the surface of the gum, along the track of a fistula.

Fig. 2

Analogous cases are by no means uncommon, though I am not aware that a case of this kind has ever been reported. One of the most interesting is described by Carl Friedlænder (vid. *Ueber Epithelwucherung u. Krebs*). He found a perfect lining of epithelium in a completely closed subcutaneous abscess. In this case the examination showed that by the suppurating process two hair follicles had been opened, and that the epithelium from the internal root membrane had gradually extended over the whole surface of the abscess. The presence of the epithelial lining, according to Friedlænder, has no effect, or if any at all, a deleterious one, upon the healing process, whether in fistulous tracks, on ulcerating or granulating surfaces, or in abscesses. The same is probably true of those perhaps rare cases where it is found in alveolar abscesses. The formation of pus takes place under the epithelial layer, just as if it were not there, and the pus corpuscles may be seen everywhere passing through the layer into the interior of the sac. We have here a case in which an atypic development of epithelium can safely be said to be without any pathological significance.

## Figures and Tables

**Fig. 1 f1:**
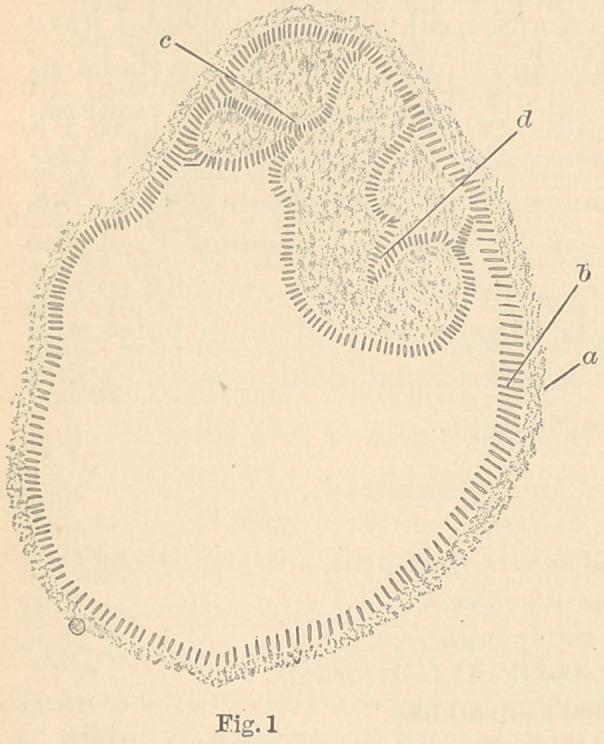


**Fig. 2 f2:**